# Long-term effects of noise pollution on the avian dawn chorus: a natural experiment facilitated by the closure of an international airport

**DOI:** 10.1098/rspb.2022.0906

**Published:** 2022-09-14

**Authors:** Léna de Framond, Henrik Brumm

**Affiliations:** Communication and Social Behaviour Group, Max Planck Institute for Ornithology, Eberhard-Gwinner-Straße, Seewiesen 82319, Germany

**Keywords:** airport, animal communication, anthropogenic noise, bird song, dawn chorus, noise pollution

## Abstract

The impacts of noise pollution on birdsong have been extensively investigated but potential long-term effects are neglected. Near airports, where noise levels are particularly high, birds start singing earlier in the morning, probably to gain more time of uninterrupted singing before air traffic sets in. In a previous study, we documented this phenomenon in the vicinity of Berlin Tegel airport. In 2020, Tegel airport closed down, giving us the opportunity to investigate potential long-term effects after noise removal and to gain insight into the mechanisms underlying the advancement of dawn singing. We found that several species at the airport shifted their song onset back after the closure and now had similar schedules to their conspecifics at a control site. Some species, however, still sang earlier near the closed airport. While the first suggests plastic adaptation, the latter suggests selection for early singing males in areas with long-lasting noise pollution. Our findings indicate that a uniform behavioural response to anthropogenic change in a community can be based on diverging evolutionary mechanisms. Overall, we show that noise pollution can have long-lasting effects on animal behaviour and noise removal may not lead to immediate recovery in some species.

## 1. Introduction

Anthropogenic noise is arguably one of the most pervasive and least controlled pollutants, with vehicle and aircraft noise being particularly widespread [1]. In the European Union, for instance, more than 100 million people are affected by hazardous traffic noise levels [2]. These hazards include sleep deprivation, hypertension and cardiovascular disease, metabolic dysregulation, psychological disorders, and reduced cognitive performance [3]. For these reasons, the World Health Organization classified traffic noise as a major threat to public health [1]. Noise is not only detrimental to humans, it also affects many non-human animals, including arthropods, fish, amphibians, birds and mammals [4]. Typically, noise impacts animals on different biological system levels, from physiology to behaviour and ecological processes [5,6]. Hence, it is of major importance to understand how noise pollution affects wildlife [7,8].

Generally, noise can have two types of effect on animals: auditory effects (i.e. impairments of hearing and masking of acoustic signals or cues, and non-auditory effects, such as stress, noise-induced diseases, and changes in predator or prey abundance). Anthropogenic noise has auditory effects in animals that use sound to communicate or to find their prey [9]. For instance, noise from traffic and industry infrastructure interferes with the detection of alarm calls by birds [10,11], which is likely to increase the predation risk in noise-polluted areas. Traffic noise also disrupts the detection of acoustic cues used by greater mouse-eared bats (*Myotis myotis*) to find their insect prey, which leads to a reduced hunting efficiency close to motorways [12]. As for non-auditory effects of anthropogenic noise, a growing body of evidence from different taxa has identified effects on stress physiology and the immune system [13,14], as well as on behaviour, including acoustic signalling [15,16], space use [17,18] and learning [19,20]. Other non-auditory effects include reduced pairing and breeding success [21,22]. Ultimately, noise pollution can affect whole communities [23–26] and alter ecological services [27]. Two recent studies found that the abundance of different bird species and their reproductive success varies with noise pollution levels across a continental scale [28,29].

In the context of noise pollution, studying animal behaviour is of special interest for two reasons. First, behaviour is the interface between the physiological changes in an animal and the environment; second, behaviour can be markedly plastic, allowing rapid adaptations to changing environments. One particular behaviour that has been widely studied in relation to anthropogenic noise is bird song [30,31]. Noise effects on bird song have strong implications for the evolution of signals as well as for conservation [32], and for almost 20 years, researchers have been investigating whether and how birds adjust their songs to anthropogenic noise. It emerges that the most basic mechanism is the regulation of vocal amplitude (the Lombard effect), which is probably present in all birds [33]. In addition, some species also adjust the timing and frequency of their songs in response to anthropogenic noise [30]. Counteracting acoustic masking is crucial for birds because their songs carry vital information. Specifically, birds use their songs in territory defence and mate attraction [34]. Therefore, differences in the efficiency of signal transmission due to noise likely have major fitness consequences.

A particularly severe case of noise pollution is that from aircraft [1]. Noise measurements in bird territories close to airport runways have registered peak levels as high as 87–118 dB(A) SPL [35,36], which is above the limit that birds can compensate through the Lombard effect [37]. Shifts in song frequency are of no help either, as aircraft noise is typically very broadband, covering the entire frequency range of bird songs [38]. On top of this, major airports often operate almost continuously throughout the day, with airplane take-offs every one to two minutes [39]. The resulting extreme noise pollution poses an unusual challenge to birds, most likely surpassing all natural noise sources they have encountered in their evolutionary past. Therefore, noise pollution from airports is not only a special concern for conservation but also an eminent case for research into the mechanisms of song adaptation.

It appears that birds in the vicinity of airports adjust their song timing in relation to the airplane noise. For instance, chaffinches (*Fringilla coelebs*) fell silent during fly-overs from starting airplanes when the noise exceeded 78 dB(A) SPL [35]. In addition to such short-term plasticity in response to single noise events, many bird species in noise-polluted areas begin singing earlier in the morning [35,40,41]. This phenomenon leads to an advancement of the so-called ‘dawn chorus’ (i.e. the marked peak of singing activity around dawn in the breeding season) by 4–45 min, depending on the species and the airport location [35,40,42]. The dawn chorus in Europe usually starts before airports begin their daily operations, and it is thought that birds at airports advance their dawn song onsets to gain more time of unimpaired singing before the onset of air traffic [35]. This shift seems crucial since singing around dawn is optimal to attract mates and defend territories [43]. It remains unknown, however, how the advancement in song onset in noise-polluted areas arises. Two hypotheses have been put forward to explain the emergence of this phenomenon: (i) population-wide, microevolutionary changes (e.g. through selection for earlier chronotypes), and (ii) behavioural plasticity (i.e. individual short-term changes in song onset in response to changes in the environment) [35,40].

The closure of the Berlin Tegel international airport in November 2020 afforded us the opportunity to test these hypotheses in a natural experiment. Tegel airport opened in 1948 as a military airport and civil aviation with regular flights started operating in 1960 [44]. Thus, the forest bordering the airport was exposed to frequent high-level noise pollution for at least 60 years, which might have led, over the course of many generations, to microevolutionary changes in the local bird populations. In a previous study, while the airport was still operating, we recorded the onset of the dawn song for all species of the bird community in a forest close to the airport and at control sites together with the environmental noise levels, and we then quantified the noise-related shift in the dawn chorus [35]. Now we intend gaining insight into the mechanisms underlying the noise-related advancement of dawn singing. To this end, we repeated the previous study during the first breeding season after the airport closure in the same areas as in the previous study. The selection hypothesis (H1) predicts that birds near the airport still sing earlier than in the control areas. The behavioural-plasticity hypothesis (H2), in contrast, predicts that birds shift back to normal dawn song schedules so that no difference in song onsets times between airport and control locations can be detected.

## 2. Methods

### (a) Field recordings

We recorded the bird dawn chorus at two forested sites, referred to as 'airport' and 'control', on 2, 3 and 4 May 2021. These sites were the same as in a previous study by Dominoni *et al*. [35]. The control forest was chosen because it was close to the airport site (the sites were roughly 4 km apart; electronic supplementary material, figure S1), and it had a similar age and vegetation structure (mixed deciduous and pine forests with little undergrowth). Within each site, recordings were made at 21 locations. To this end, we used 14 AudioMoth audio recorders (v. 1.2.0) [45], seven of which were deployed at each site at the same time, and then swapped between locations the next day. The audio recorders were packed in resealable plastic bags to protect them from humidity and then attached to trees. The locations were chosen so that the surface area of both sites was well covered but the recorded areas did not overlap (based on previous tests, we estimated the recording distance of each unit to be around 100 m). Each recording (sample rate 32 kHz, gain ‘medium’) started at 03.40 and lasted until 06.30, resulting in 170 files with a duration of 55 s, separated by a 5 s pause (we chose to split up the recording into short files because they are easier to handle, the 5 s pause was necessary to allow the system to save the data on the SD card without overloading the memory).

All recordings were analysed with Avisoft-SASLab Pro software (v. 5.2.08, Avisoft Bioacoustics, Berlin, Germany) by the same observer (LdF). For every recording session (one recording unit, 1 day), the spectrograms (FFT window 256, gain 30) were visually screened until the first bird vocalization was detected and then all following files were listened to. Species songs (or drumming in case of the great spotted woodpecker) were identified and the onset time (minute at which the first bird of each species was heard) was noted. This scoring was done blindly (i.e. the observer was not informed about the site of the recording when identifying the species). To verify that the scoring in the present study was comparable with that of Dominoni *et al*. [35], one recording session was also analysed by one of the observers involved in the previous study (HB). Both observers detected the same 21 species, for 18 of which they had an inter-observer reliability for the dawn chorus onset of 100%, for two species the detected onset time differed by 1 min, and for one species it differed by 2 min.

In addition to the onset of the dawn chorus, we also used the Audiomoth recordings to measure the ambient noise levels. For this purpose, one 55 s file per location was chosen between 06.15 and 06.30. We selected this time period because it is the noise levels after 06.00 that were crucial for the advancement of the dawn chorus at Tegel airport [35]. For the noise level measurements, we selected recordings with no wind and no birds singing close to the recorder. We bandpass filtered the recordings in the range of bird hearing (0.1–10.0 kHz), then corrected them for the frequency response of the microphone and finally applied an A weighting (see 'Recorder calibration' below). Similarly to Dominoni *et al*. [35], we define ambient levels as the sound level (dB(A) RMS re 20 µPa) of the 100 ms window with the highest value in the selected 55 s file.

### (b) Recorder calibration

To obtain accurate sound level measurements, it is necessary to correct the recordings for the frequency response of the recording system because microphones do not record all frequencies with the same amplitude. Therefore, we measured the frequency response and the sensitivity of each recorder in the range of bird hearing. All sound generation and analyses for the calibration were performed in R (v. 4.0.4, R Foundation for Statistical Computing) with the package *seewave* (v. 2.1.6) [46]. The calibration was done separately for each audio recorder.

We generated a pulse train (100 Hz–10 kHz in 100 Hz steps, pulse duration 0.2 s including a 0.05 s linear fade-in and 0.05 s linear fade-out) and a 10 s 1 kHz tone. This playback was broadcasted through a Pioneer A-109 amplifier and a JBP Pro III loudspeaker and then recorded with an AudioMoth recorder and at the same time with a Behringer ECM 8000 measuring microphone (connected to a Marantz PMD 660 recorder). The source level of the 1 kHz tone was measured with a Casella CEL-240 SPL meter. The AudioMoth recorder, the measuring microphone, and the SPL meter were mounted 1 m in front of the loudspeaker in an anechoic room, the floor and walls of which were covered with sound-absorbing foam. The frequency response of the loudspeaker was first calculated using the recordings made with the measuring microphone. The central section of each pulse (0.08 s excluding the fade-in and the fade-out) was extracted from the recordings and then bandpass filtered ±200 Hz around the pulse frequency. Thereafter, we calculated the amplitude of each pulse (dB RMS FS). In a next step, we subtracted the amplitude of the 1 kHz pulse from the amplitude values obtained for all other frequencies, such that the amplitude of all pulses is expressed in dB relative to the amplitude of the 1 kHz signal. This procedure was applied to each audio recorder used in this study. We then subtracted the frequency profile of the loudspeaker (measured with the measuring microphone) from the frequency profile obtained for the audio recorders, to obtain the frequency response of each individual recorder. We padded zeros before 100 Hz and after 10 kHz and performed a linear interpolation on the frequency response to obtain 256 values, equally spaced between 0 and 16 kHz, and added the A-weighting factor to the frequency response. We used A-weighting because it is a good proxy for the frequency-dependant sensitivity of bird hearing [47]. The frequency response was then used as an impulse-response filter. The received level of the 1 kHz tone (dB RMS FS) was used to determine the sensitivity of each recording unit. Based on the sensitivity and frequency response curves, we could then obtain the true ambient sound levels from the recordings.

### (c) Statistical analysis

Statistical analyses were conducted in R (v. 4.0.4), using the package *lme4* (v. 1.1-26) and *arm* (v. 1.11-2). In line with our previous study [35], we included in the analysis all species that were detected at least at ten different locations at each site. To compare the effect of the site (airport or control) on the onset of dawn chorus before the airport closure [35] with the situation after the closure (present study), we performed a similar analysis as described in [35]. We fitted a multiple linear regression with the onset time (in minutes after civil twilight) as the response variable for all species together (global model). The peak ambient level measured from the recordings, and the site (airport versus control) were included as fixed predictors. The date (3 May, 4 May and 5 May) was also included as a fixed predictor to account for potential day-to-day variability in singing activity independent of noise levels and site (due, e.g. to differences in the weather). The species was included as a random factor to account for species-specific variability in the singing behaviour. The recorder ID was used as a random factor to account for potential differences in recording quality. We checked model fit by visual inspection of the diagnostic plots [48] (i.e. we made sure that residuals and random effects were normally distributed, residuals plotted against fitted values did not show any signs of heteroscedasticity or any obvious trend, and that there were no autocorrelations in the residuals). Credible intervals of estimates were obtained by simulating the posterior distribution of the model 1000 times and calculating the 2.5% and 97.5% percentiles of the simulated estimates [49]. In addition to the global model, we also analysed the effect of the site for each species separately because previous studies have found species-specific effects of the ambient noise on dawn chorus onset times [35,40,50]. For this purpose, we fitted 15 sub-models (one per species) with the date, ambient noise level and the site as predictors of the onset of dawn chorus, and with the recorder ID as a random effect. We used the same procedure as described for the global model to check model fit and to calculate credible intervals for each of the 15 species-specific models. Altogether, we constructed 16 different models that investigated the long-term effect of noise pollution on the onset of the avian dawn chorus: one global model, across all species, and 15 species-specific models. Because our aim was to compare the onset of the dawn chorus before and after the closure of the airport, we refitted the species-specific models with the data from [35] to obtain the respective estimates and credible intervals.

## 3. Results

After the closure of the airport, the median peak level of environmental noise at the airport site was 46.2 dB(A), which is a drop by more than 28 dB(A) compared to the noise levels when the airport was operating (two sample *t*-test: 95% confidence interval = −33.69, −28.34; *p* < 0.001). Still, the airport locations were on average somewhat noisier than the control locations (two sample *t*-test: 95% confidence interval = 1.41, 9.02; *p* = 0.008), but this difference was as little as 3.9 dB(A) (electronic supplementary material, figure S2).

In total, we recorded 46 species in the dawn chorus recordings, 45 at the airport site and 33 at the control site (electronic supplementary material, table S1). Of these, 15 species were detected more than 10 times at both sites, including all of the 10 species analysed in the previous study when the airport was still operating. The order in which the different species started singing around dawn was similar across all recording locations (electronic supplementary material, figure S3).

The global model indicated that birds near the airport started the dawn chorus on average 3.83 min earlier compared to birds in the control forest (table 1). However, unlike in the previous study, the onset of the dawn chorus did not vary with ambient noise levels (table 1). The day of the recordings had also no significant effect on chorus onset times (table 1).

**Table 1.** Estimates, credible intervals and s.e. of the general linear mixed model explaining the dawn chorus onset time across all species (global model). The intercept represents the average onset time on 2 May at the control site. The ‘site’ variable shows the effect of the airport site relative to the control site. Statistically significant variables are shown in italics.

	estimate (95% CRI)	s.e.	*t*-value
(intercept)	26.62 (13.51, 39.01)	6.53	4.11
*site*	*−3.83 (−6.71, −0.96)*	*1.46*	*−2.66*
peak ambient level	−0.11 (−0.36, 0.14)	0.12	−0.85
date 3 May	−0.75 (−3.87, 2.24)	1.58	−0.49
date 4 May	1.28 (−2.01, 4.31)	1.65	0.76

**Figure 1.**
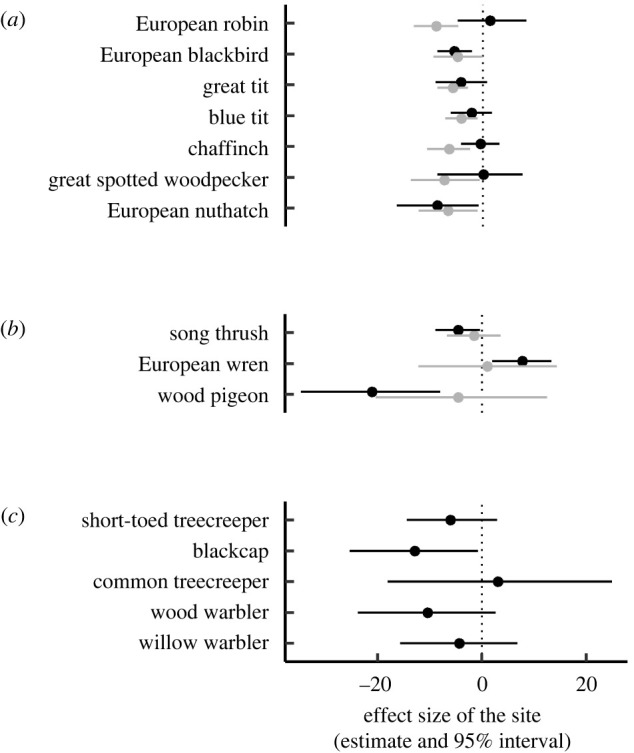
Effect sizes (average and 95% credible interval) of the difference in the onset of dawn song between the airport site and the control site. The dotted line indicates no effect of the site, i.e. birds start singing at the same time in both forests. Negative values indicate earlier song onsets at the airport than in the control forest and positive values indicate later song onsets at the airport than in the control forest. Grey: airport operating (spring 2013 and 2014); black: airport closed (spring 2021). Species are organized in three categories based on their behaviour when the airport was operating [35]: (*a*) bird species that sang significantly earlier at the airport, (*b*) bird species that did not sing significantly earlier at the airport and (*c*) bird species that were not investigated when the airport was operating.

While the global model points to a persisting effect of the airport site on the onset of the dawn chorus after the airport was closed (table 1), our species-specific analyses show that the birds’ reactions to the closure of the airport differed between species. The bird species that we considered in our analyses fall into three categories: the species that started the dawn chorus significantly earlier at the airport site when the airport was operating (seven species, figure 1*a*), the species that did not sing significantly earlier at the airport when it was operating (three species; figure 1*b*), and the species that were not analysed in the previous study because they occurred at less than ten locations per site but have now passed this threshold after the closure of the airport (five species; figure 1*c*).

Of the seven species in the first category that commenced the dawn chorus earlier at the airport site while it was operating (figure 1*a*), five shifted the chorus onset to later times after the airport was closed down, namely robins, great tits, blue tits, chaffinches and great spotted woodpeckers. The effect sizes in the two tit species were larger (greater than 2 min) than in the other three species (less than 2 min, credible intervals centred on zero) and they fell in-between zero and the values measured while the airport was operating. Blackbirds and nuthatches still sang considerably earlier at the airport site compared to the control site (effect size greater than 5 min, credible interval not overlapping with 0), just as they did when the airport was in operation (figure 1*a*, table 2*a*).

**Table 2.** Estimates, credible intervals and s.e. of the species-specific linear mixed models explaining the dawn chorus onset time as a function of the site. The intercept represents the average onset time on 2 May at the control site. The ‘site’ variable shows the effect of the airport relative to the control. Species are organized in three categories based on their behaviour when the airport was operating [35]: (*a*) bird species that sang significantly earlier at the airport, (*b*) bird species that did not sing significantly earlier at the airport and (*c*) bird species that were not investigated when the airport was operating. Statistically significant variables are shown in italics.

species		estimate (95% CRI)	s.e.	*t*-value
(*a*) species that sang earlier at the airport while it was operating				
European robin	(intercept)	−28.26 (−35.01; −21.87)	3.71	−7.59
	site	1.37 (−4.8; 8.3)	3.82	0.4
	*date 3 May*	*16.97 (9.56; 24.46)*	*4.5*	*3.71*
	date 4 May	12.37 (4.24; 20.61)	4.8	2.57
European blackbird	(Intercept)	−0.47 (−3.9; 2.78)	2	−0.19
	*site*	*−5.44 (−8.68; −2.1)*	*1.97*	*−2.77*
	date 3 May	0.69 (−3.21; 4.59)	2.36	0.25
	date 4 May	0.47 (−3.73; 4.85)	2.45	0.12
great tit	(intercept)	11.24 (6.48; 16.05)	2.89	3.94
	site	−4.17 (−9.05; 0.78)	2.93	−1.43
	date 3 May	−1.52 (−7.13; 4.24)	3.47	−0.49
	date 4 May	1.89 (−4.25; 7.9)	3.68	0.43
blue tit	(intercept)	17.7 (13.99; 21.44)	2.27	7.76
	site	−2.18 (−6.16; 1.75)	2.34	−0.87
	date 3 May	2.29 (−2.23; 7.18)	2.8	0.81
	date 4 May	−3.09 (−8.08; 2)	2.87	−1.07
chaffinch	(intercept)	21.61 (17.89; 25.22)	2.08	10.4
	site	−0.43 (−4.17; 3.12)	2.13	−0.21
	*date 3 May*	*−4.47 (−8.73; −0.41)*	*2.53*	*−1.78*
	date 4 May	−0.29 (−4.74; 3.95)	2.64	−0.15
great spotted woodpecker	(intercept)	38.67 (31.02; 46.59)	4.61	8.37
	site	0.1 (−8.74; 7.6)	4.75	0.09
	date 3 May	4.76 (−4.7; 14.07)	5.67	0.82
	date 4 May	0.54 (−9.04; 9.98)	5.81	0.07
European nuthatch	(intercept)	53.95 (45.65; 62.22)	4.8	11.19
	*site*	*−8.69 (−16.5; −0.8)*	*4.65*	*−1.87*
	date 3 May	2.29 (−6.84; 12.27)	5.65	0.49
	date 4 May	−10.08 (−19.48; −0.62)	5.77	−1.75
(*b*) species that did not sing earlier at the airport while it was operating				
song thrush	(intercept)	−5.78 (−10.11; −1.24)	2.49	−2.37
	*site*	*−4.58 (−8.96; −0.36)*	*2.54*	*−1.8*
	date 3 May	−4.34 (−9.41; 0.62)	3.04	−1.4
	date 4 May	0.48 (−4.98; 5.61)	3.18	0.23
European wren	(intercept)	6.3 (0.41; 12.39)	3.32	1.86
	*site*	*7.72 (1.92; 13.28)*	*3.4*	*2.3*
	date 3 May	−5.88 (−12.95; 1.22)	4.15	−1.41
	date 4 May	−0.67 (−7.55; 6.36)	4.05	−0.16
wood pigeon	(intercept)	49.5 (36.46; 61.88)	7.41	6.67
	*site*	*−21.03 (−34.71; −7.99)*	*7.69*	*−2.71*
	date 3 May	8.48 (−5.75; 22.17)	8.71	0.98
	*date 4 May*	*18.72 (2.15; 35.84)*	*9.96*	*1.9*
(*c*) new species that were not analysed while the airport was operating				
short-toed treecreeper	(intercept)	25.99 (17.59; 34.72)	5.18	5.01
	site	−6.01 (−14.42; 2.92)	5.34	−1.17
	date 3 May	2.16 (−8.55; 13.06)	6.42	0.39
	date 4 May	−4.81 (−15.87; 6.22)	6.45	−0.72
blackcap	(intercept)	42.37 (28.27; 56.18)	8.19	5.22
	*site*	*−12.88 (−25.38; −0.71)*	*7.65*	*−1.71*
	date 3 May	−7.05 (−21.68; 8.44)	9.03	−0.8
	date 4 May	1.51 (−15.73; 18.42)	9.86	0.15
common treecreeper	(intercept)	41.26 (21.75; 60.92)	11.32	3.64
	site	3.11 (−18.07; 24.93)	12.05	0.28
	date 3 May	−8.53 (−34.61; 18.45)	15.79	−0.57
	date 4 May	−0.3 (−22.34; 24.06)	13.56	0
wood warbler	(intercept)	72.49 (59.67; 85.52)	7.71	9.36
	site	−10.38 (−23.79; 2.65)	7.88	−1.28
	*date 3 May*	*−17.47 (−34.67; −0.45)*	*9.87*	*−1.79*
	date 4 May	−4.81 (−21.56; 12.26)	9.51	−0.47
willow warbler	(intercept)	69.22 (59.18 79)	5.95	11.66
	site	−4.34 (−15.71; 6.83)	6.36	−0.72
	date 3 May	−8.47 (−21.58; 3.18)	7.25	−1.18
	date 4 May	1.69 (−11.8; 16.18)	8.35	0.18

The species in the second category (those that did not sing significantly earlier in the presence of noise) shifted their dawn song onsets in different directions after the noise removal (figure 1*b*): song thrushes and woodpigeons started singing considerably earlier at the airport (on average 4.6 and 21.1 min, respectively; table 2*b*), whereas wrens started singing later at the airport compared to the control area (on average 7.7 min).

Finally, in the third category (figure 1*c*), four of the five species that were not included in the previous study [35] tended to sing earlier at the airport compared to the control site although the noise pollution had been removed for almost six months (mean effect size between 4 and 12 min; table 2*c*). It is important to note that the sample sizes in this group of species was smaller than in the other two categories (electronic supplementary material, table S1) and probably because of this the variation in the data resulted in wide credible intervals (that overlapped zero in the short-toed treecreeper, the wood warbler and the willow warbler), calling for a careful interpretation of the results.

## 4. Discussion

Evidence for the impact of anthropogenic noise on animals is growing [6,28,51] but only few studies have examined potential long-term effects. Birds advance the onset of their diel singing activity in areas that are heavily noise polluted during the day [40–42], and we hypothesized that this is either the result of behavioural plasticity or the outcome of selection for earlier chronotypes [35]. Here, we used the opportunity of the closure of an international airport to test these hypotheses. We found that most species at the airport shifted their song onsets back after the closure and had now similar dawn song schedules as their conspecifics in a control forest. However, some species still started singing earlier in the vicinity of the airport and a general trend of earlier dawn song onsets at the airport could still be detected across the entire bird community (table 1).

Thus, we found support for both the selection (H1) and the behavioural-plasticity hypothesis (H2). In line with H1, blackbirds, nuthatches, song thrushes, wood pigeons and blackcaps still sang earlier at the airport after the closure (figure 1). There is ample evidence that environmental selection through noise may shape acoustic signals, resulting in population-wide changes in signal characteristics in many taxa (reviewed in [52–54]). For instance, grasshoppers from noisy road-side habitats produce mating songs with elevated frequencies that are less masked by the vehicle noise and this increased song frequency persist when the insects are transferred to a silent room [55]. Moreover, there is a cross-generational effect of the noise, as the offspring from road-side grasshoppers also produce higher-pitched songs, even when they are reared with no noise exposure [16]. Our study suggests that not only the signal itself but also when it is produced can be subjected to more permanent shifts in chronically noisy environments. Such a long-term shift may be based on selection for certain chronotypes [35]. Several studies have shown that the timing of song onset and other behaviours can be under sexual selection [56–58]. Likewise, the timing of dawn song could be under environmental selection, with the massive noise pollution from aircraft leading to the selection of males with earlier song onsets. Such a scenario would explain the patterns we observed in the species that still sang earlier at the airport although noise pollution had stopped (e.g. song thrush, blackbird and nuthatch; figure 1). If the observed persistence of the advanced song timing indeed reflects selection for earlier chronotypes, then we would expect that these species will return only slowly to later song onsets at the silent airport site, probably over the course of several generations.

By contrast, robins, great tits, blue tits and chaffinches had shifted back their song onsets at the airport (figure 1), suggesting noise-dependant plasticity of dawn song timing in these species. Likewise, great spotted woodpeckers started drumming later in the morning at the airport after it had been closed, resulting in similar daily routines as their conspecifics in the control forest. Thus, the onset of drumming in woodpeckers appears to be as plastic as the dawn song in some songbird species and, just as well, modulated by the level of noise masking later in the day. Noise-dependant song plasticity is well documented in birds (reviewed in [30]). Presumably all extant birds exhibit the Lombard effect (i.e. they increase their vocal amplitude when background noise levels rise) [33]. In addition, some species may also adjust song pitch [59] or song rate [60] in response to anthropogenic noise. Spotless starlings (*Sturnus unicolor*) and house sparrows (*Passer domesticus*) shifted their dawn chorus onset on a daily basis when they were experimentally exposed to traffic noise [61], which is in line with the behavioural-plasticity hypothesis. Similarly, most species in our study shifted the onset of their dawn chorus to later schedules after the noise pollution from the airport ceased, corroborating the notion of noise-related song plasticity. Sierro *et al*. [42] suggested that the advanced blackbird dawn chorus at airports is also a plastic adjustment, as the observed birds shifted song onsets only early in the season, when the dawn chorus overlapped with aircraft noise at their study site in Spain. However, the two studies conducted at Tegel airport indicate that the dawn chorus in blackbirds was affected by long-term effects of noise pollution. Blackbirds near the operating airport began the dawn chorus significantly earlier even though the song onset did not overlap with aircraft noise (which set in about 70 min later) [35], and they still sang earlier six months after the closure of the airport (present study). Taken together, these findings support the selection hypothesis rather than the behavioural-plasticity hypothesis for this species. Conflicting results from different locations may be accounted for by latitudinal differences in the onset of the dawn chorus and, related to this, in the resulting response to noise pollution, as suggested by Gil *et al*. [40]. Indeed, no consistent dawn chorus shift could be found in bird communities around tropical airports [50]. These differences between tropical and temperate birds suggest that biogeography can have substantial effects on how animals respond to anthropogenic change [41].

Although the exact mechanism underlying the observed behavioural plasticity in our study is not known, the results indicate that some bird species are able to anticipate the onset of noise masking later in the day and to flexibly adjust their song onset accordingly. In a classic experiment, Gwinner [62] demonstrated that social sound cues can function as zeitgeber for circadian rhythms in songbirds, in particular, he found that Eurasian siskins (*Spinus spinus*) and European serins (*Serinus serinus*) synchronize their daily activity patterns to the periodic broadcast of conspecific song. Our findings suggest that other periodic sound cues, such as anthropogenic noise, can have similar effects on the chronobiology of at least some bird species.

In addition to noise-induced microevolutionary shifts and song plasticity, the onset of dawn song may also be affected indirectly by the massive noise pollution, such as through changes in the predatory landscape. It is known that Passerines sing more and earlier when the perceived predation pressure is low [63]. Moreover, anthropogenic noise can disrupt both the distribution [23] and the hunting success [64] of predators. Therefore, heavy noise pollution might lead to reduced predation pressure on birds and, in turn, result in advanced song onset. On the other hand, anthropogenic noise can also mask the alarm calls of songbirds [10] which then increases predation risk. Without empirical data, however, it is impossible to tell what the outcome of these opposing factors is, and it remains to be shown whether the potential noise-induced changes in predation indeed affect the onset of the dawn chorus.

While there is increasing interest in the impacts of anthropogenic noise on wildlife [65], potential long-term effects have been neglected. One notable exception comes from the work by Clinton Francis and colleagues on the ecological impacts of noise from gas well compressors in New Mexico. These compressors emit continuous noise at high amplitudes, which has strong effects on the behaviour of birds and mammals, leading to large-scale modifications in plant communities through altered seed dispersal and pollination [27]. In some areas, the compressors had been switched off (after running for a decade or so) but the plant community did not recover within the first four years after the noise removal [66]. This long-term disruption is the outcome of cascading ecological effects, in which the negative impact of noise pollution may persist for longer periods than in our study that addressed behavioural responses of individual animals. However, our results indicate that noise pollution can also have long-lasting effects on individual behaviours in some species.

After Tegel airport was shut down, the noise levels in the adjacent forest dropped massively as expected. It must be noted, though, that even after the closure the ambient noise was slightly higher at the airport site than the control forest. However, the average ambient noise level at the airport locations was 46 dB(A) SPL, which is within the range of natural noise levels in a temperate forest [30,67]. Moreover, the mean difference in noise levels between the airport and the control site was lower than 4 dB, which is unlikely to affect the song timing. In previous studies, shifts in the dawn chorus were related to much larger noise differences, namely 8–30 dB [61], 20–25 dB [40] and 30 dB [35]. Indeed, our global model indicated no effect of the noise level on the onset of dawn chorus. Therefore, we are confident that the observed advances in dawn singing near the airport in our study were due to carry-over effects of the noise pollution from previous years rather than the current differences in ambient noise levels.

In conclusion, our study suggests that intense anthropogenic noise pollution can have long-term consequences for animal behaviour, even after noise emissions have ceased. Specifically, we still observed advanced dawn singing six months after an international airport stopped operating, which means that birds did not shift their behavioural routines back to normal times after the massive noise pollution from aircraft was removed. On the other hand, some species quickly shifted their song onset back to the typical schedule of undisturbed conditions, illustrating the complexity of noise pollution impacts on wildlife. Our study indicates that both phenotypic plasticity and population-wide long-term changes may lead to a noise-induced advance of dawn chorus onsets in different species. A better understanding of the long-term consequences of pollution on organisms and ecosystems is of major importance for conservation so that mitigation and avoidance measures can be implemented to minimize not only immediate but also long-term impacts.

## Data Availability

The data used in this study are available from the Open Research Data Repository of the Max Planck Society (https://doi.org/10.17617/3.EGLBLP) [68] and in the electronic supplementary material [69].

## References

[1] World Health Oorganization. 2011 Burden of disease from environmental noise: quantification of healthy life years lost in Europe. Copenhagen, Denmark: World Health Organization Regional Office for Europe.

[2] European Environment Agency. 2020 Environmental noise in Europe—2020. Luxembourg, Luxembourg: Publications Office of the European Union.

[3] Basner M, Babisch W, Davis A, Brink M, Clark C, Janssen S, Stansfeld S. 2014 Auditory and non-auditory effects of noise on health. Lancet **383**, 1325-1332. (10.1016/S0140-6736(13)61613-X) 24183105 PMC3988259

[4] Kunc HP, Schmidt R. 2019 The effects of anthropogenic noise on animals: a meta-analysis. Biol. Lett. **15**, 20190649. (10.1098/rsbl.2019.0649) 31744413 PMC6892517

[5] Barber JR, Crooks KR, Fristrup KM. 2010 The costs of chronic noise exposure for terrestrial organisms. Trends Ecol. Evol. **25**, 180-189. (10.1016/j.tree.2009.08.002) 19762112

[6] Kight CR, Swaddle JP. 2011 How and why environmental noise impacts animals: an integrative, mechanistic review. Ecol. Lett. **14**, 1052-1061. (10.1111/j.1461-0248.2011.01664.x) 21806743

[7] Francis CD, Barber JR. 2013 A framework for understanding noise impacts on wildlife: an urgent conservation priority. Front. Ecol. Environ. **11**, 305-313. (10.1890/120183)

[8] Jerem P, Mathews F. 2021 Trends and knowledge gaps in field research investigating effects of anthropogenic noise. Conserv. Biol. **35**, 115-129. (10.1111/cobi.13510) 32277776

[9] McGregor P, Horn AG, Leonard ML, Thomsen F. 2013 Anthropogenic noise and conservation. In Animal communication and noise (ed. H Brumm), pp. 409-444. Berlin, Germany: Springer.

[10] Templeton CN, Zollinger SA, Brumm H. 2016 Traffic noise drowns out great tit alarm calls. Curr. Biol. **26**, R1173-R1174. (10.1016/j.cub.2016.09.058) 27875691

[11] Antze B, Koper N. 2018 Noisy anthropogenic infrastructure interferes with alarm responses in Savannah sparrows (*Passerculus sandwichensis*). R. Soc. Open Sci. **5**, 172168. (10.1098/rsos.172168) 29892404 PMC5990837

[12] Siemers BM, Schaub A. 2011 Hunting at the highway: traffic noise reduces foraging efficiency in acoustic predators. Proc. R. Soc. B **278**, 1646-1652. (10.1098/rspb.2010.2262) PMC3081776 21084347

[13] Tennessen JB, Parks SE, Swierk L, Reinert LK, Holden WM, Rollins-Smith LA, Walsh KA, Langkilde T. 2018 Frogs adapt to physiologically costly anthropogenic noise. Proc. R. Soc. B **285**, 20182194. (10.1098/rspb.2018.2194) PMC6253376 30464067

[14] Zollinger SA, Dorado-Correa A, Goymann W, Forstmeier W, Knief U, Bastidas-Urrutia AM, Brumm H. 2019 Traffic noise exposure depresses plasma corticosterone and delays offspring growth in breeding zebra finches. Conserv. Physiol. **7**, coz056. (10.1093/conphys/coz056) 31620292 PMC6788579

[15] Brumm H. 2004 The impact of environmental noise on song amplitude in a territorial bird. J. Anim. Ecol. **73**, 434-440. (10.1111/j.0021-8790.2004.00814.x)

[16] Lampe U, Reinhold K, Schmoll T. 2014 How grasshoppers respond to road noise: developmental plasticity and population differentiation in acoustic signalling. Funct. Ecol. **28**, 660-668. (10.1111/1365-2435.12215)

[17] McClure CJW, Ware HE, Carlisle J, Kaltenecker G, Barber JR. 2013 An experimental investigation into the effects of traffic noise on distributions of birds: avoiding the phantom road. Proc. R. Soc. B **280**, 20132290. (10.1098/rspb.2013.2290) PMC3826227 24197411

[18] Luo JH, Siemers BM, Koselj K. 2015 How anthropogenic noise affects foraging. Glob. Change Biol. **21**, 3278-3289. (10.1111/gcb.12997) 26046451

[19] Brumm H, Goymann W, Deregnaucourt S, Geberzahn N, Zollinger SA. 2021 Traffic noise disrupts vocal development and suppresses immune function. Sci. Adv. **7**, eabe2405. (10.1126/sciadv.abe2405) 33980481 PMC8115921

[20] Osbrink A et al. 2021 Traffic noise inhibits cognitive performance in a songbird. Proc. R. Soc. B **288**, 20202851. (10.1098/rspb.2020.2851) PMC7893234 33529564

[21] Habib L, Bayne EM, Boutin S. 2007 Chronic industrial noise affects pairing success and age structure of ovenbirds *Seiurus aurocapilla*. J. Appl. Ecol. **44**, 176-184. (10.1111/j.1365-2664.2006.01234.x)

[22] Francis CD, Paritsis J, Ortega CP, Cruz A. 2011 Landscape patterns of avian habitat use and nest success are affected by chronic gas well compressor noise. Landsc. Ecol. **26**, 1269-1280. (10.1007/s10980-011-9609-z)

[23] Francis CD, Ortega CP, Cruz A. 2009 Noise pollution changes avian communities and species interactions. Curr. Biol. **19**, 1415-1419. (10.1016/j.cub.2009.06.052) 19631542

[24] McClure CJW, Ware HE, Carlisle JD, Barber JR. 2017 Noise from a phantom road experiment alters the age structure of a community of migrating birds. Anim. Conserv. **20**, 164-172. (10.1111/acv.12302)

[25] Willems JS, Phillips JN, Francis CD. 2022 Artificial light at night and anthropogenic noise alter the foraging activity and structure of vertebrate communities. Sci. Total Environ. **805**, 150223. (10.1016/j.scitotenv.2021.150223) 34537710

[26] Bayne EM, Habib L, Boutin S. 2008 Impacts of chronic anthropogenic noise from energy-sector activity on abundance of songbirds in the boreal forest. Conserv. Biol. **22**, 1186-1193. (10.1111/j.1523-1739.2008.00973.x) 18616740

[27] Francis CD, Kleist NJ, Ortega CP, Cruz A. 2012 Noise pollution alters ecological services: enhanced pollination and disrupted seed dispersal. Proc. R. Soc. B **279**, 2727-2735. (10.1098/rspb.2012.0230) PMC3367785 22438504

[28] Senzaki M et al. 2020 Sensory pollutants alter bird phenology and fitness across a continent. Nature **587**, 605-609. (10.1038/s41586-020-2903-7) 33177710

[29] Wilson AA, Ditmer MA, Barber JR, Carter NH, Miller ET, Tyrrell LP, Francis CD. 2021 Artificial night light and anthropogenic noise interact to influence bird abundance over a continental scale. Glob. Change Biol. **27**, 3987-4004. (10.1111/gcb.15663) 34111313

[30] Brumm H, Zollinger SA. 2013 Avian vocal production in noise. In Animal communication and noise (ed. H Brumm), pp. 187-227. Berlin, Germany: Springer.

[31] Dooling RJ, Blumenrath SH. 2013 Avian sound perception in noise. In Animal communication and noise (ed. H Brumm), pp. 229-250. Berlin, Germany: Springer.

[32] Gil D, Brumm H. 2014 Acoustic communication in the urban environment: patterns, mechanisms, and potential consequences of avian song adjustments. In Avian urban ecology (eds D Gil, H Brumm), pp. 69-83. Oxford, UK: Oxford University Press.

[33] Brumm H, Zollinger SA. 2011 The evolution of the Lombard effect: 100 years of psychoacoustic research. Behaviour **148**, 1173-1198. (10.1163/000579511X605759)

[34] Catchpole CK, Slater PJB. 2008 Bird song. Biological themes and variations, 2nd edn. Cambridge, UK: Cambridge University Press.

[35] Dominoni DM, Greif S, Nemeth E, Brumm H. 2016 Airport noise predicts song timing of European birds. Ecol. Evol. **6**, 6151-6159. (10.1002/ece3.2357) 27648232 PMC5016638

[36] Wolfenden AD, Slabbekoorn H, Kluk K, de Kort SR. 2019 Aircraft sound exposure leads to song frequency decline and elevated aggression in wild chiffchaffs. J. Anim. Ecol. **88**, 1720-1731. (10.1111/1365-2656.13059) 31435938 PMC8647924

[37] Nemeth E, Brumm H. 2010 Birds and anthropogenic noise: are urban songs adaptive? Am. Nat. **176**, 465-475. (10.1086/656275) 20712517

[38] Asensio C, Pavon I, Ruiz M, Pagan R, Recuero M. 2007 Estimation of directivity and sound power levels emitted by aircrafts during taxiing, for outdoor noise prediction purpose. Appl. Acoust. **68**, 1263-1279. (10.1016/j.apacoust.2006.07.014)

[39] Gatwick Airport Ltd. 2018 Gatwick Airport Flight Performance Team Annual Report 2017. See https://www.gatwickairport.com/business-community/aircraft-noise-airspace/noise-reports/. Retrieved 12.12.2021.

[40] Gil D, Honarmand M, Pascual J, Perez-Mena E, Garcia CM. 2015 Birds living near airports advance their dawn chorus and reduce overlap with aircraft noise. Behav. Ecol. **26**, 435-443. (10.1093/beheco/aru207)

[41] Dorado-Correa AM, Rodriguez-Rocha M, Brumm H. 2016 Anthropogenic noise, but not artificial light levels predicts song behaviour in an equatorial bird. R. Soc. Open Sci. **3**, 160231. (10.1098/rsos.160231) 27493778 PMC4968470

[42] Sierro J, Schloesing E, Pavon I, Gil D. 2017 European blackbirds exposed to aircraft noise advance their chorus, modify their song and spend more time singing. Front. Ecol. Evol. **5**, 68. (10.3389/fevo.2017.00068)

[43] Gil D, Llusia D. 2020 The dawn chorus revisited. In Coding strategies in vertebrate acoustic communication (eds T Aubin, N Mathevon), pp. 45-90. Cham, Switzerland: Springer.

[44] Berlin Airport Company. 1985 Special Report on Air France's 25th Anniversary at Berlin Tegel. In Monthly timetable booklet for Berlin Tegel airport. Berlin (West), Germany: Berlin Airport Company.

[45] Hill AP, Prince P, Covarrubias EP, Doncaster CP, Snaddon JL, Rogers A. 2018 AudioMoth: evaluation of a smart open acoustic device for monitoring biodiversity and the environment. Methods Ecol. Evol. **9**, 1199-1211. (10.1111/2041-210X.12955)

[46] Sueur J, Aubin T, Simonis C. 2008 Seewave, a free modular tool for sound analysis and synthesis. Bioacoustics **18**, 213-226. (10.1080/09524622.2008.9753600)

[47] Dooling RJ, Lohr B, Dent ML. 2000 Hearing in birds and reptiles. In Comparative hearing: birds and reptiles (eds RJ Dooling, RR Fay, AN Popper), pp. 308-359. New York, NY: Springer.

[48] Pinheiro J, Bates D. 2006 Mixed-effects models in S and S-PLUS. New York, NY: Springer.

[49] Abbey-Lee RN, Kaiser A, Mouchet A, Dingemanse NJ. 2016 Immediate and carry-over effects of perceived predation risk on communication behavior in wild birds. Behav. Ecol. **27**, 708-716. (10.1093/beheco/arv210)

[50] Alquezar RD, Macedo RH, Sierro J, Gil D. 2020 Lack of consistent responses to aircraft noise in dawn song timing of bird populations near tropical airports. Behav. Ecol. Sociobiol. **74**, 88. (10.1007/s00265-020-02865-6)

[51] Simpson SD, Radford AN, Nedelec SL, Ferrari MCO, Chivers DP, McCormick MI, Meekan MG. 2016 Anthropogenic noise increases fish mortality by predation. Nat. Commun. **7**, 10544. (10.1038/ncomms10544) 26847493 PMC4748250

[52] Brumm H, Naguib M. 2009 Environmental acoustics and the evolution of bird song. Adv. Stud. Behav. **40**, 1-33. (10.1016/S0065-3454(09)40001-9)

[53] Brumm H. 2013 Animal communication and noise. Berlin, Germany: Springer.

[54] Wiley H. 2015 Noise matters: the evolution of communication. Cambridge, MA: Harvard University Press.

[55] Lampe U, Schmoll T, Franzke A, Reinhold K. 2012 Staying tuned: grasshoppers from noisy roadside habitats produce courtship signals with elevated frequency components. Funct. Ecol. **26**, 1348-1354. (10.1111/1365-2435.12000)

[56] Poesel A, Kunc HP, Foerster K, Johnsen A, Kempenaers B. 2006 Early birds are sexy: male age, dawn song and extrapair paternity in blue tits, *Cyanistes* (formerly *Parus*) *caeruleus*. Anim. Behav. **72**, 531-538. (10.1016/j.anbehav.2005.10.022)

[57] Greives TJ et al. 2015 Costs of sleeping in: circadian rhythms influence cuckoldry risk in a songbird. Funct. Ecol. **29**, 1300-1307. (10.1111/1365-2435.12440)

[58] Hau M, Dominoni D, Casagrande S, Buck CL, Wagner G, Hazlerigg D, Greives T, Hut RA. 2017 Timing as a sexually selected trait: the right mate at the right moment. Phil. Trans. R. Soc. B **372**, 20160249. (10.1098/rstb.2016.0249) 28993493 PMC5647276

[59] Bermúdez-Cuamatzin E, Ríos-Chelén AA, Gil D, Garcia CM. 2011 Experimental evidence for real-time song frequency shift in response to urban noise in a passerine bird. Biol. Lett. **7**, 36-38. (10.1098/rsbl.2010.0437) 20610421 PMC3030866

[60] Potvin DA, Parris KM, Mulder RA. 2011 Geographically pervasive effects of urban noise on frequency and syllable rate of songs and calls in silvereyes (*Zosterops lateralis*). Proc. R. Soc. B **278**, 2464-2469. (10.1098/rspb.2010.2296) PMC3125619 21208948

[61] Arroyo-Solis A, Castillo JM, Figueroa E, Lopez-Sanchez JL, Slabbekoorn H. 2013 Experimental evidence for an impact of anthropogenic noise on dawn chorus timing in urban birds. J. Avian Biol. **44**, 288-296. (10.1111/j.1600-048X.2012.05796.x)

[62] Gwinner E. 1966 Periodicity of a circadian rhythm in birds by species-specific song cycles (Aves, Fringillidae: *Carduelis spinus*, *Serinus serinus*). Experientia **22**, 765-766. (10.1007/BF01901370)

[63] Schmidt KA, Belinsky KL. 2013 Voices in the dark: predation risk by owls influences dusk singing in a diurnal passerine. Behav. Ecol. Sociobiol. **67**, 1837-1843. (10.1007/s00265-013-1593-7)

[64] Senzaki M, Yamaura Y, Francis CD, Nakamura F. 2016 Traffic noise reduces foraging efficiency in wild owls. Sci. Rep. **6**, 30602. (10.1038/srep30602) 27537709 PMC4989872

[65] Shannon G et al. 2016 A synthesis of two decades of research documenting the effects of noise on wildlife. Biol. Rev. **91**, 982-1005. (10.1111/brv.12207) 26118691

[66] Phillips JN, Termondt SE, Francis CD. 2021 Long-term noise pollution affects seedling recruitment and community composition, with negative effects persisting after removal. Proc. R. Soc. B **288**, 20202906. (10.1098/rspb.2020.2906) PMC8059579 33849312

[67] Lawrence BT, Hornberg J, Haselhoff T, Sutcliffe R, Ahmed S, Moebus S, Gruehn D. 2022 A widened array of metrics (WAM) approach to characterize the urban acoustic environment; a case comparison of urban mixed-use and forest. Appl. Acoust. **185**, 108387. (10.1016/j.apacoust.2021.108387)

[68] Brumm H, de Framond L. 2022 Dataset for: Long-term effects of noise pollution on the avian dawn chorus: a natural experiment facilitated by the closure of an international airport. Edmond. (10.17617/3.EGLBLP)

[69] de Framond L, Brumm H. 2022 Long-term effects of noise pollution on the avian dawn chorus: a natural experiment facilitated by the closure of an international airport. FigShare. (10.6084/m9.figshare.c.6168339) PMC9470256 36100015

